# Induction of a novel isoform of the lncRNA HOTAIR in Claudin‐low breast cancer cells attached to extracellular matrix

**DOI:** 10.1002/1878-0261.12133

**Published:** 2017-10-30

**Authors:** Miao Li, Xi Li, Yan Zhuang, Erik K. Flemington, Zhen Lin, Bin Shan

**Affiliations:** ^1^ Department of Microbiology and Parasitology College of Basic Medical Sciences China Medical University Shenyang China; ^2^ Department of Biomedical Sciences Elson S. Floyd College of Medicine Washington State University Spokane WA USA; ^3^ Department of Sports Medicine and Joint Surgery The People's Hospital of Liaoning Province Shenyang China; ^4^ Department of Medicine Tulane University School of Medicine New Orleans LA USA; ^5^ Department of Pathology Tulane University School of Medicine New Orleans LA USA

**Keywords:** epigenetics, HOTAIR, lncRNA, three‐dimensional organotypic culture

## Abstract

Elevated overexpression of the lncRNA HOTAIR mediates invasion and metastasis in breast cancer. In an apparent paradox, we observed low expression of HOTAIR in the invasive Claudin‐low MDA‐MB‐231 and Hs578T cells in two‐dimensional culture (2D). However, HOTAIR expression exhibited robust induction in laminin‐rich extracellular matrix‐based three‐dimensional organotypic culture (lrECM 3D) over that in 2D culture. Induction of HOTAIR required intact ECM signaling, namely integrin α2 and SRC kinase activity. Moreover, invasive growth was suppressed by HOTAIR‐specific siRNA. Induction of HOTAIR in lrECM 3D culture resulted from the activation of a novel isoform of HOTAIR (HOTAIR‐N) whose transcription is started from the first intron of the HOXC11 gene. The HOTAIR‐N promoter exhibited increased trimethylation of histone H3 lysine 4, a histone marker of active transcription, and binding of BRD4, a reader of transcriptionally active histone markers. Inhibition of BRD4 substantially reduced the expression of HOTAIR in lrECM 3D culture. In summary, our results indicate that HOTAIR expression is activated by BRD4 binding to a novel HOTAIR‐N promoter in Claudin‐low breast cancer cells that are attached to ECM. Induction of HOTAIR is required for invasive growth of Claudin‐low breast cancer cells in lrECM 3D culture.

**Abbreviations**
BRD4: bromodomain containing 4
ECM: extracellular matrix
H3K4me3: trimethylation of histone H4 lysine 4
HOTAIR: HOX transcript antisense RNA
HOX: homeobox
lrECM: laminin‐rich extracellular matrix
TSA: trichostatin A

## 1 Introduction

Human breast cancer is classified into ‘intrinsic subtypes’: luminal A, luminal B, basal, Claudin‐low, and HER2‐enriched based on gene expression profiles (Perou *et al*., 2000; Prat and Perou, 2011; Sorlie *et al*., 2001). This molecular stratification complements the pathological classifiers of breast cancer, namely estrogen receptor (ER), progesterone receptor (PR), and human epidermal growth factor receptor 2 (HER2), and is preserved in the established human breast cancer cell lines (Holliday and Speirs, 2011; Parker *et al*., 2009; Prat and Perou, 2011). Claudin‐low subtype correlates with triple‐negative (ER‐negative, PR‐negative, and HER2‐negative) invasive ductal carcinomas (Holliday and Speirs, 2011; Prat *et al*., 2010). The gene expression profile of Claudin‐low subtype is enriched with the signaling components that regulate cellular responses to extracellular matrix (ECM) (Charafe‐Jauffret *et al*., 2009; Creighton *et al*., 2009; Hennessy *et al*., 2009; Prat *et al*., 2010; Shipitsin *et al*., 2007; Taube *et al*., 2010). This feature implies that attachment to ECM has profound impacts on gene expression of Claudin‐low breast cancer cells. Laminin‐rich ECM (Matrigel) three‐dimensional organotypic culture (lrECM 3D), pioneered by Bissell's group, is an ideal model system to investigate ECM‐regulated gene expression because it faithfully recapitulates salient properties of breast cancer cells attached to ECM (Kenny *et al*., 2007a). Moreover, the gene signatures of breast cancer cells in lrECM 3D culture hold prognostic values for patients with breast cancer (Kenny *et al*., 2007a; Martin *et al*., 2008).

One family of novel epigenetic regulators of gene expression is long noncoding RNA (lncRNA) (Rinn and Chang, 2012). The lncRNA genes exhibit tissue‐specific expression patterns and regulate expression of the genes that are pivotal to development and cancer (Cabili *et al*., 2011; Guttman *et al*., 2009). lncRNA can act as a recruiter and scaffold for assembly of chromatin modifiers on their target genes (Chu *et al*., 2011; Gupta *et al*., 2010; Spitale *et al*., 2011; Tsai *et al*., 2010; Wang and Chang, 2011). The lncRNA HOX transcript antisense RNA (HOTAIR) is elevated in breast cancer and promotes metastasis (Gupta *et al*., 2010; Rinn *et al*., 2007). HOTAIR recruits polycomb repressive complex 2 (PCR2) to their target genes for transcriptional repression (Chu *et al*., 2011). In accordance, an EMT‐like derivative of the human breast cancer MCF‐7 cells exhibited a robust increase in HOTAIR expression and such an increase was required for accelerated proliferation and resistance to cell death (Antoon *et al*., 2012; Zhuang *et al*., 2015). One intriguing observation in the discovery of HOTAIR in breast cancer is that the RNA levels of HOTAIR in breast cell lines in cell culture are significantly lower than those observed in primary and metastatic breast tumors (Gupta *et al*., 2010). This observation implies that conventional 2D culture lacks critical factors that stimulate the expression of HOTAIR. One critical factor absent in 2D culture is attachment to ECM. Our recent report suggests a link between collagen and HOTAIR in lung adenocarcinoma cells (Zhuang *et al*., 2013).

Emerging evidence from lrECM 3D culture suggests epigenetic regulation of gene expression by the ECM signaling in cancer cells (Lelievre, 2010; Li *et al*., 2016; Teoh‐Fitzgerald *et al*., 2014). However, ECM‐regulated expression and functions of lncRNA have not been examined in Claudin‐low breast cancer cells. Herein, we aimed to understand epigenetic regulation of the lncRNA HOTAIR by ECM in Claudin‐low breast cancer cells using lrECM 3D culture.

## 2 Materials and methods

### 2.1 Reagents and plasmids

Corning (Bedford, MA, USA) provided Matrigel and Cell Recovery Solution for the extraction of cells from lrECM 3D culture. Trichostatin (TSA), a HDAC inhibitor, was purchased from Cayman Chemical (Ann Arbor, MI, USA). PP2, an Src‐specific inhibitor, was purchased from Calbiochem (San Diego, CA, USA). JQ1, a bromodomain containing 4 (BRD4)‐specific inhibitor, was a kind gift from James Bradner at Dana‐Farber Cancer Institute (Loven *et al*., 2013). The GAPDH‐ and BRD4‐specific antibodies were purchased from Novus Biologicals (Littleton, CO, USA) and Cell Signaling (Danvers, MA, USA), respectively. An integrin α2β1‐neutralizing antibody (clone JBS2) was purchased from Chemicon (Temecula, CA, USA) (Knight *et al*., 1998). A retroviral vector expressing dominant‐negative chicken Src‐K295R mutant (dnSrc) was kindly provided by Joan Brugge at Harvard University (Thomas *et al*., 1991).

### 2.2 Cell lines and cell culture

Two Claudin‐low human breast cancer cell lines MDA‐MB‐231 and Hs578T were purchased from ATCC (Manassas, VA, USA) (Holliday and Speirs, 2011). The cell lines were cultured in Dulbecco's modified Eagle's medium (DMEM) as we previously described (Zhuang *et al*., 2010).

### 2.3 lrECM 3D organotypic culture

Overlay lrECM 3D culture was carried out as described elsewhere (Li *et al*., 2011; Vidi *et al*., 2013). Briefly, MDA‐MB‐231 and Hs578T cells were seeded at a density of 2 × 10^5 ^cells per well in a six‐well culture dish that was coated with Matrigel. DMEM culture medium supplemented with 4% of Matrigel was replaced every two days. The morphology of cell clusters was monitored and recorded using an inverse phase contrast microscope linked with a digital camera and fluorescent staining for filamentous actin using Alexa 488‐conjugated phalloidin followed by confocal microscopy (Li *et al*., 2011).

### 2.4 Retroviral transduction

The integrin α2‐specific Mission shRNA lentiviral transduction particles and its matching control were purchased from Sigma (St. Louis, MO, USA). Retroviral transduction was carried out as described elsewhere (Shan *et al*., 2007). The stably transduced MDA‐MB‐231 cells were selected using puromycin. In a similar fashion, we generated the MDA‐MB‐231 variants that stably expressed a dominant‐negative Src mutant chicken Src‐K295R (Nguyen *et al*., 2013; Thomas *et al*., 1991).

### 2.5 Transient transfection and RNA interference

Sigma provided the human BRD4‐specific Mission siRNA (BRD4siRNA, SIHK0192, SIHK0193, SIHK0194), the HOTAIR‐specific Mission siRNA (HOTAIRsiRNA, Sigma ID SASI_Hs02_00380445), and the control Mission siRNA. The HOTAIR isoform NR_047517 (HOTAIR‐N)‐specific siRNA were designed using IDT's siRNA designing tool and purchased from IDT (Coralville, IA, USA). The sequences of the HOTAIR‐N‐specific siRNA were provided in Table S1. All the siRNA were transfected at 60 nm into MDA‐MB‐231 and Hs578T cells using RNAiMAX per the reverse transfection protocol (Invitrogen, Carlsbad, CA, USA; Shan *et al*., 2005). Total RNA and protein were extracted on day 3 after transfection in 2D culture and day 4 after transfection in lrECM 3D culture.

### 2.6 Cell viability assay

Cell viability in lrECM 3D culture was measured using XTT *in vitro* Toxicology Assays (Sigma) as we previously described (Shan and Morris, 2005). The values from the control groups were set to 100%.

### 2.7 RNA extraction and RT‐PCR

Total cell RNA was extracted using TRIzol (Invitrogen) from 2D and lrECM 3D cultures at the indicated time points as previously described (Li *et al*., 2012). Quantitative RT‐PCR (qRT‐PCR) was carried out to determine the RNA levels of the genes of interest. Each transcript was normalized to a housekeeping gene ribosomal protein large P0 (RPLP0). A fold change of each transcript was obtained by setting the values from the control groups to one.

### 2.8 Immunoblot

Total cell lysates were extracted from MDA‐MB‐231 cells exposed to the indicated treatments using 1× Laemmli buffer. In lrECM 3D culture, MDA‐MB‐231 cells were separated from Matrigel using BD cell recover solution as previously described (Nguyen *et al*., 2013). Immunoblotting was used to measure the protein levels of integrin α2, BRD4, and GAPDH (Shan *et al*., 2010).

### 2.9 Chromatin immunoprecipitation

Chromatin immunoprecipitation (ChIP) was performed as previously described with minor modifications (Li *et al*., 2016). Millipore (Darmstadt, Germany) provided EZ ChIP Kit (Cat #17–371). The ChIP‐grade H3K4me3 antibody was purchased from Active Motif (Carlsbad, CA, USA). Sheared chromatin was prepared from roughly 1 × 10^7^ cells using Cell Recovery Solution and then immunoprecipitated using the BRD4‐ or H3K4me3‐specific antibody, or a control antibody. The sequences of the primers specific for the human HOTAIR promoter are provided in Table S1. The input and immunoprecipitated HOTAIR promoter were quantified using qPCR. The ratios of the immunoprecipitated DNA versus its corresponding input were compared between the groups.

### 2.10 RNA‐Seq analysis

Raw RNA‐sequencing (RNA‐Seq) reads from invasive breast carcinoma samples and their paired normal samples generated through The Cancer Genome Atlas (TCGA) project were obtained from the National Cancer Institute Genomic Data Commons. The datasets were then analyzed using the RSEM algorithm for the quantification of isoforms of HOTAIR transcripts as previously described (Li and Dewey, 2011; Strong *et al*., 2014).

### 2.11 Statistical analysis

When presented, means and standard deviations were obtained from at least three independent experiments. A *P* value between any two compared groups was determined using unpaired two‐tailed Student's *t*‐test (GraphPad Prism, version 5, GraphPad Software, Inc., La Jolla, CA USA).

## 3 Results

### 3.1 Induction of the lncRNA HOTAIR in lrECM 3D culture of Claudin‐low breast cancer cells

**Figure 1:**
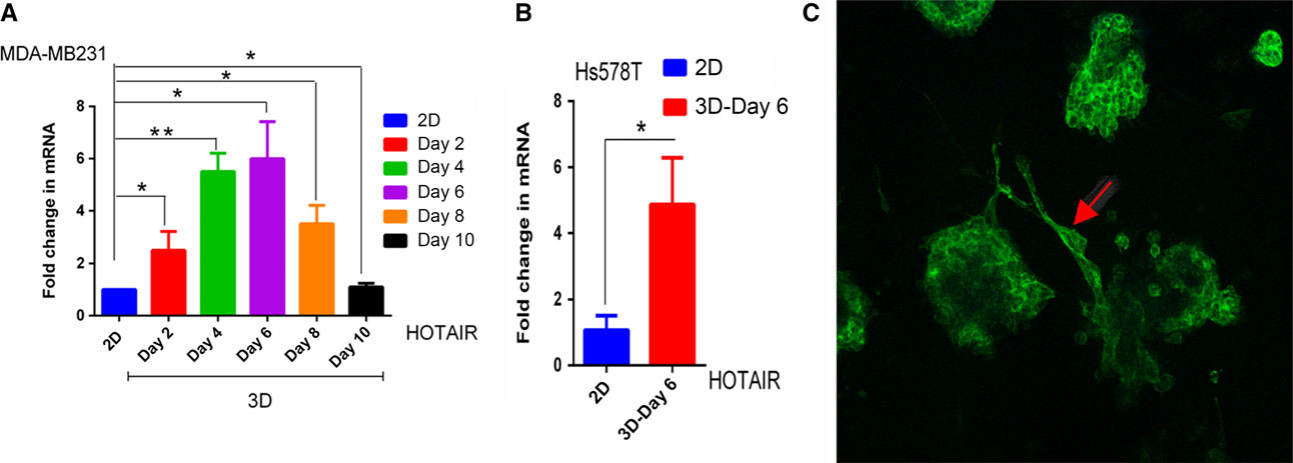
Induction of HOTAIR in lrECM 3D Culture. (A) Total cell RNA was extracted from MDA‐MB‐231 cells in 2D (day 3) and lrECM 3D cultures at the indicated time points. The RNA levels of HOTAIR were compared between two culture conditions using qRT‐PCR. A fold change of HOTAIR at each time point in lrECM 3D culture over 2D culture was obtained by normalizing to the housekeeping gene RPLP0 and setting the values from 2D culture to one. (B) Similar to part (A) except that the RNA levels of HOTAIR were compared between two culture conditions in Hs578T cells at the indicated time point. (C) The morphology of Hs578T cells in lrECM 3D culture (day 6) was visualized by staining for filamentous actin using Alexa 488‐conjugated phalloidin (pseudocolored in green). The stellate projections were indicated by red arrowheads. The image was captured at 200× magnification using a confocal microscope. * and ** indicated a *P* value < 0.05 and 0.01, respectively.

We set out to explore ECM‐mediated regulation of expression of the lncRNA HOTAIR in Claudin‐low breast cancer cells using lrECM 3D culture (Kenny *et al*., 2007b; Prat *et al*., 2010). We chose Claudin‐low MDA‐MB‐231 and Hs578T cells because of their invasive and metastatic competence (Neve *et al*., 2006; Prat *et al*., 2010). We recently reported a widespread induction of the homeobox (HOX) genes in lrECM 3D culture over 2D culture of MDA‐MB‐231 cells and Hs578T cells (Li *et al*., 2016). The HOXC cluster‐derived HOTAIR exhibited a robust increase in MDA‐MB‐231 cells from day 2 to day 10 (Fig. 1A). A similar induction of HOTAIR was observed in Hs578T cells on day 6 (Fig. 1B). Induction of HOTAIR was also observed in gene expression profiling (GEO GSE36953) of lrECM 3D culture of MDA‐MB‐231 (Yotsumoto *et al*., 2013). Induction of HOTAIR correlated with invasive growth of MDA‐MB‐231 cells and Hs578T cells (Fig. 1C) (Li *et al*., 2016). We previously reported invasive growth as evidenced by stellate morphology of MDA‐MB231 (Li *et al*., 2016). Herein, we demonstrated similar stellate morphology of Hs578T cells in lrECM 3D culture using fluorescent staining for filamentous actin (Fig. 1C). Stellate morphology of both cell lines featured irregular cell clusters and projections formed by chains of cells that intersected occasionally as described elsewhere (Fig. 1C, indicated by red arrowheads) (Kenny *et al*., 2007b).

**Figure 2:**
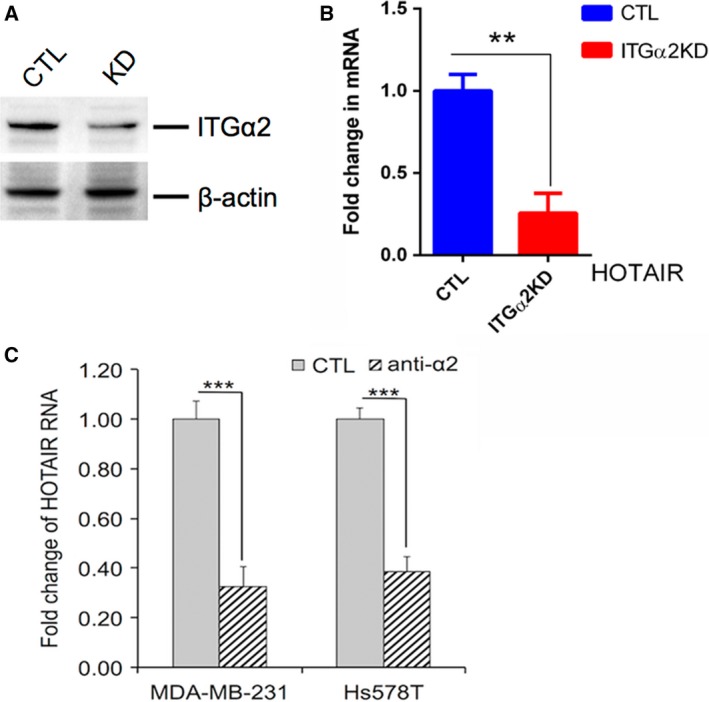
Reduced expression of HOTAIR by Inhibition of integrin α2. (A) Total cell lysates were extracted from two MDA‐MB‐231 cell variants that were transduced with lentiviral particles expressing either integrin α2‐specific Mission shRNA (ITGα2KD) or a matching control shRNA (CTL). The protein levels of integrin α2 (ITGα2) were assessed using immunoblots. (B) Total cell RNA was extracted from the ITGα2KD and CTL variants on day 6 of lrECM 3D culture of MDA‐MB‐231 cells. The RNA levels of HOTAIR were measured using qRT‐PCR. A fold change of HOTAIR in ITGα2KD cells over CTL cells was obtained by normalizing to the housekeeping gene RPLP0 and setting the values from CTL cells to one. (C) Culture conditions were similar to part (B) except that MDA‐MB‐231 and Hs578T cells were exposed to integrin α2‐neutralizing antibody (anti‐α2, 10 μg·mL^−1^) or a control antibody (CTL) in lrECM 3D culture for 6 days. ** and *** indicate a *P* value < 0.01 and 0.001, respectively.

We questioned whether induction of HOTAIR required ECM signaling in lrECM 3D culture. To this end, we generated an MDA‐MB‐231 variant in which integrin α2, a major cell surface receptor for ECM, was knocked down by the stably expressed integrin α2‐specific shRNA (ITGα2KD). The protein levels of integrin α2 were substantially reduced in ITGα2KD when compared with a matching control variant (CTL) (Fig. 2A). We measured the RNA levels of HOTAIR in ITGα2KD and CTL variants in lrECM 3D culture using qRT‐PCR. The RNA levels of HOTAIR in the ITGα2KD variant were reduced to 26% of that in the CTL variant (Fig. 2B). To confirm an essential role of integrin α2 in the induction of HOTAIR, we inhibited integrin α2 using its neutralizing antibody (clone JBS2) in lrECM 3D culture of MDA‐MB‐231 and Hs578T cells (Knight *et al*., 1998). The integrin α2‐neutralizing antibody (10 μg·mL^−1^) reduced the RNA levels of HOTAIR to 32% and 38% of that in the control IgG‐treated MDA‐MB‐231 and Hs578T cells, respectively (Fig. 2C).

**Figure 3:**
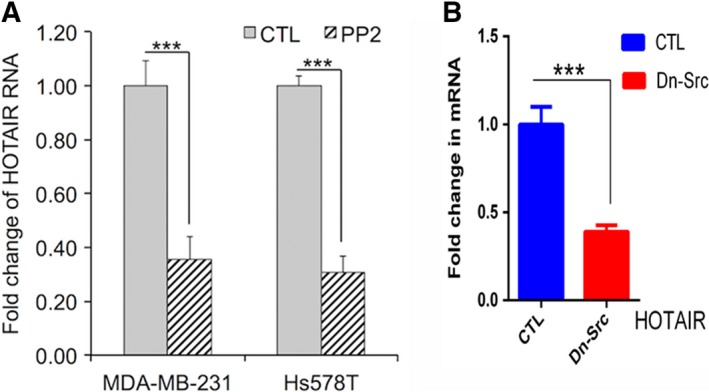
Reduced expression of HOTAIR by Inhibition of Src. (A) MDA‐MB‐231 and Hs578T cells were exposed to PP2 (5 μm) in lrECM 3D culture for 6 days. Total cell RNA was extracted from the treated cells and the RNA levels of HOTAIR were measured using quantitative RT‐PCR. A fold change of HOTAIR was obtained by normalizing to the housekeeping gene RPLP0 and setting the values from CTL to one. (B) Total cell RNA was extracted from the MDA‐MB‐231 variants expressing dominant‐negative Src (Dn‐Src) or its vector control (CTL) in lrECM 3D culture on day 6. The RNA levels of HOTAIR were measured and compared as in part (A). *** indicated a *P* value < 0.001.

Src kinase is a key intracellular signal transducer downstream of integrins in response to ECM and growth factors in lrECM 3D culture (Huang *et al*., 2011; Nguyen *et al*., 2013). Thus, we questioned whether Src kinase activity is required for the induction of HOTAIR in lrEMC 3D culture. To this end, we exposed MDA‐MB‐231 and Hs578T cells to PP2 (5 μm), an Src‐specific inhibitor in lrECM 3D culture. PP2 reduced the RNA levels of HOTAIR to 36% and 31% of the DMSO groups in MDA‐MB‐231 and Hs578T cells, respectively (Fig. 3A). To further confirm a requirement of Src kinase activity for the induction of HOTAIR in lrECM 3D culture, we generated two variants of MDA‐MB‐231 cells that were transduced with either a retroviral vector expressing a dominant‐negative Src mutant (MDA‐MB‐231‐dnSrc) or its backbone vector (CTL). As expected, the RNA levels of HOTAIR in MDA‐MB‐231‐dnSrc were reduced to 39% of that in the control group (Fig. 3B). These findings indicated that HOTAIR was induced in lrECM 3D culture via the ECM signaling.

### 3.2 Requirement of HOTAIR for invasive growth of Claudin‐low breast cancer cells in lrECM 3D culture

**Figure 4:**
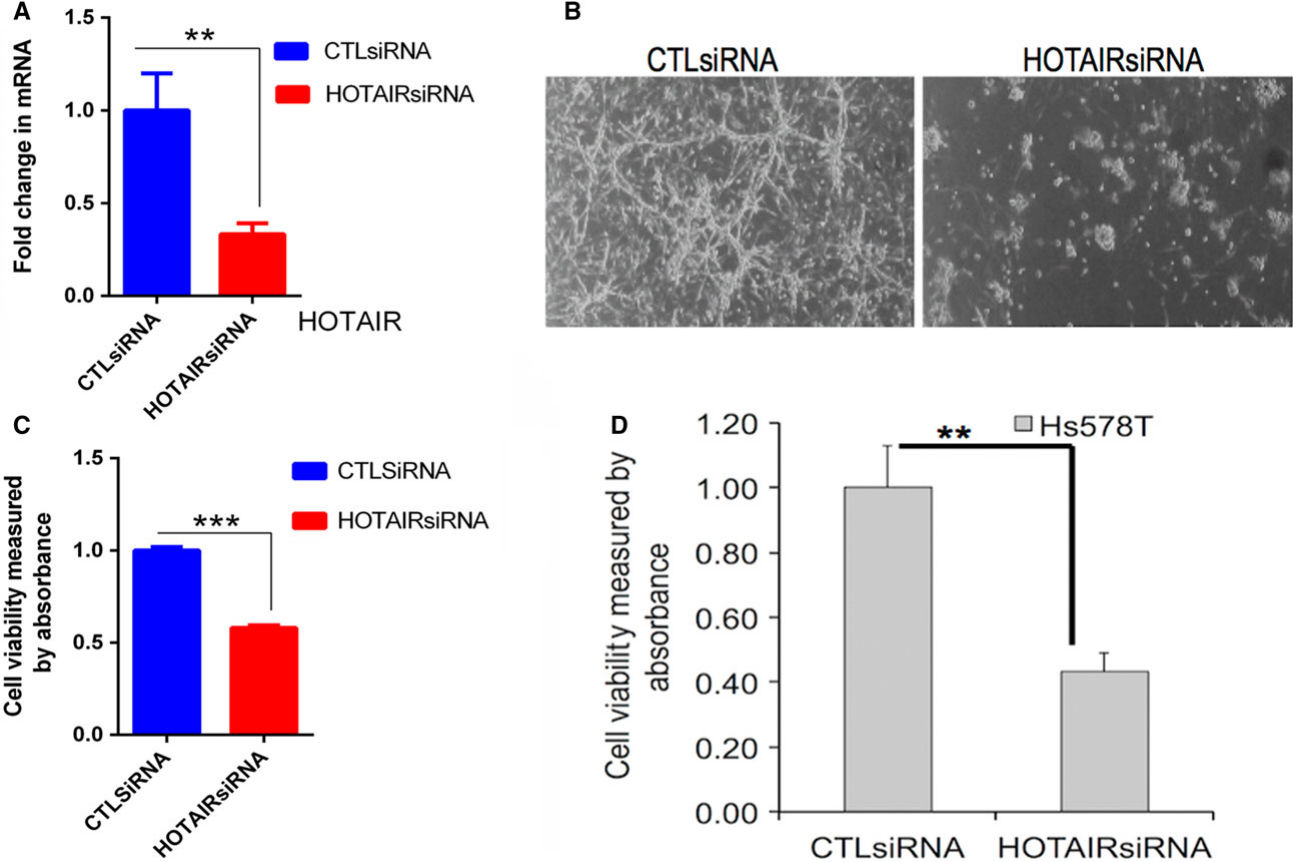
Requirement of HOTAIR for invasive growth of Claudin‐low breast cancer cells. (A) MDA‐MB‐231 cells were transfected with either a control siRNA (CTLsiRNA) or a HOTAIR‐specific siRNA (HOTAIRsiRNA). The RNA levels of HOTAIR were measured using qRT‐PCR. A fold change was obtained by normalizing to the housekeeping gene RPLP0 and setting the values from the CTLsiRNA group to one. (B) Growth pattern was monitored in lrECM 3D culture of MDA‐MB‐231 cells transfected with either HOTAIRsiRNA or CTLsiRNA using a phase contrast microscope linked to a digital camera. The images were taken on day 6 in lrECM 3D culture. (C) Culture conditions were similar to part (B) except that cell viability was measured using XTT assays in MDA‐MB‐231 cells. A fold change was obtained by setting the values from CTLsiRNA group to one. (D) Similar to part (C) except that cell viability was measured in Hs578T cells. ** and *** indicated a *P* value < 0.01 and 0.001, respectively.

Claudin‐low MDA‐MB‐231 and Hs578T cells exhibited invasive growth in lrECM 3D culture (Fig. 1C; Kenny *et al*., 2007b; Li *et al*., 2016). A correlation between induction of HOTAIR and invasive growth prompted us to examine the role of HOTAIR in invasive growth in lrECM 3D culture. We transfected MDA‐MB‐231 cells with either the HOTAIR‐specific siRNA (HOTAIRsiRNA) or control siRNA (CTLsiRNA). RNAi‐mediated knockdown of HOTAIR was confirmed by a decrease to 33% of the control transfection (Fig. 4A). In accordance, the HOTAIRsiRNA‐transfected group was void of invasive growth pattern, whereas the CTLsiRNA‐transfected group exhibited invasive growth (Fig. 4B). We also noticed an apparent decrease in number of cells (Fig. 4B). Thus, we examined cell viability of MDA‐MB‐231 and Hs578T cells transfected with either the HOTAIRsiRNA or CTLsiRNA using XTT assays. The HOTAIRsiRNA‐transfected groups exhibited a substantial decrease in cell viability, to 58% in MDA‐MB‐231 and 43% in Hs578T cells, respectively (Fig. 4C,D).

### 3.3 Induction of HOTAIR‐N in lrECM 3D culture of Claudin‐low breast cancer cells

**Figure 5:**
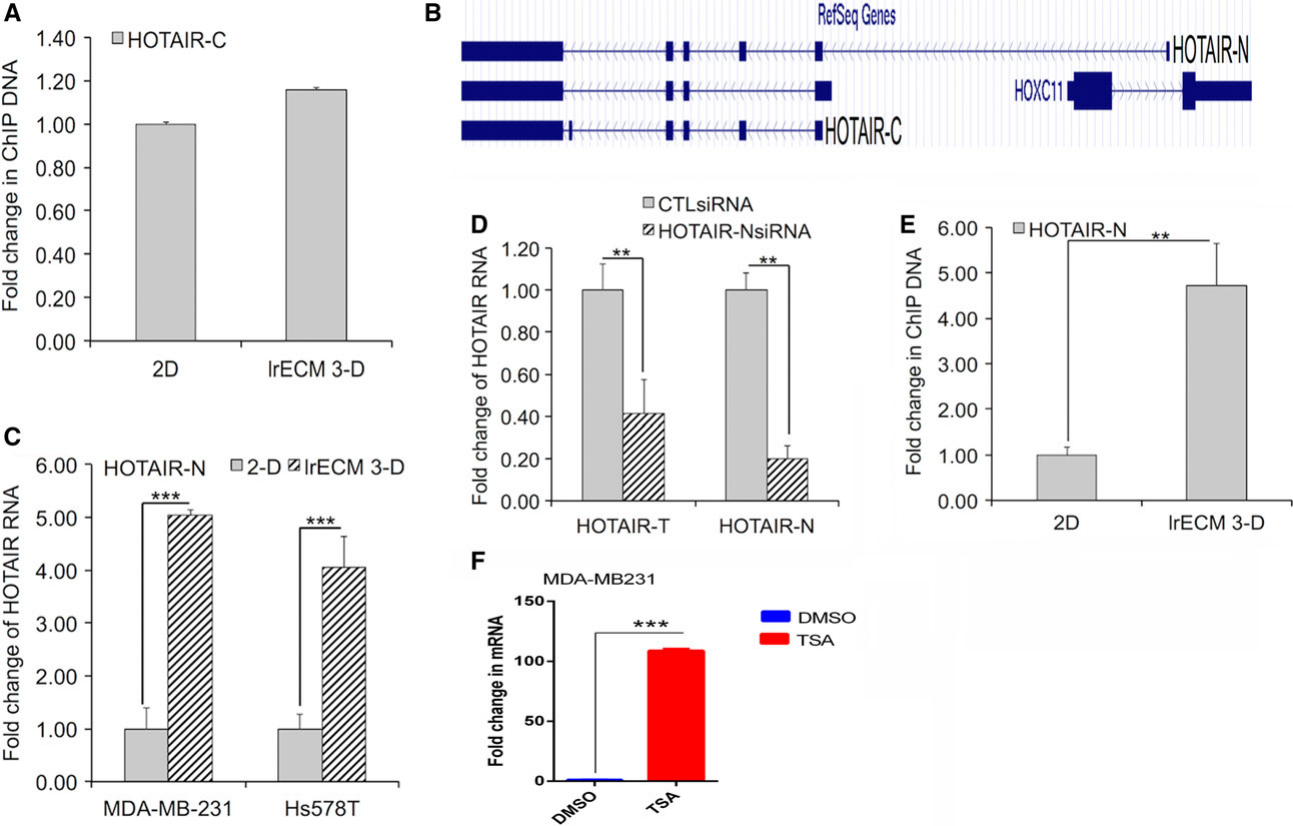
Induction of HOTAIR‐N in lrECM 3D Culture. (A) ChIP assays were carried out using an H3k4me3‐specific antibody in 2D and lrECM 3D cultures of MDA‐MB‐231 cells on day 4. The H3K4me3‐associated promoter of the canonical HOTAIR transcript (HOTAIR‐C) was measured using qPCR and normalized to their corresponding input. A fold change of the H3K4me3‐associated HOTAIR‐C promoter in lrECM 3D culture over 2D culture was obtained by setting the values from 2D culture to one. (B) A visual presentation of the HOTAIR isoforms in RefSeq using UCSC Genome Browser. (C) Total cell RNA was extracted from 2D and lrECM 3D cultures of MDA‐MB‐231 and Hs578T cells. The RNA levels of the novel HOXC11‐overlapping HOTAIR transcript (HOTAIR‐N) were measured using qRT‐PCR. A fold change was obtained by normalizing to the housekeeping gene RPLP0 and setting the values from 2D culture to one. (D) MDA‐MB‐231 cells were transfected with either a control siRNA (CTLsiRNA) or a HOTAIR‐N‐specific siRNA (HOTAIR‐NsiRNA). Similar to part (C), the RNA levels of total HOTAIR (HOTAIR‐T) and HOTAIR‐N were measured using qRT‐PCR. (E) Similar to part (A) except that the H3K4me3‐associated promoter of the HOTAIR‐N transcript (HOTAIR‐N) was assessed using ChIP assays. (F) MDA‐MB‐231 cells were exposed to TSA (500 nm) for 72 h in 2D culture. The RNA levels of HOTAIR were assessed using qRT‐PCR. A fold change was obtained by normalizing to the housekeeping gene RPLP0 and setting the values from DMSO group to one. ** and *** indicated a *P* value < 0.01 and 0.001, respectively.

We speculated that induction of HOTAIR expression resulted from activation of the HOTAIR promoter in lrECM 3D culture. We initially focused on the promoter of the canonical transcript NR_003716 (HOTAIR‐C) that was first discovered in breast cancer (Gupta *et al*., 2010; Rinn *et al*., 2007). We examined trimethylation of histone 3 lysine 4 (H3K4me3) that has been used as a marker of the transcriptionally active lncRNA (Guttman *et al*., 2009). To our surprise, we observed minimal difference in the promoter region proximal to the transcription start site of HOTAIR‐C (−287 to −404 relative to the transcription initiation site) between 2D and lrECM 3D cultures (Fig. 5A). We were aware that the primers for qRT‐PCR can amplify all three HOTAIR isoforms as listed in the latest RefSeq release 81 (Fig. 5B) (O'Leary *et al*., 2016). Thus, induction of HOTAIR in lrECM 3D culture could result from the activation of a novel isoform other than HOTAIR‐C. We speculated that NR_047517 (HOTAIR‐N) and its promoter accounted for the induction of HOTAIR in lrECM 3D culture of Claudin‐low breast cancer cells because its transcription start site is located in the first intron of the HOXC11 gene and distant from the promoters for the other two HOTAIR isoforms (Fig. 5B). We examined the RNA levels of HOTAIR‐N by qRT‐PCR using a pair of HOTAIR‐N‐specific primers. In congruence to induction of HOTAIR using the nonisoform‐specific primers, we observed a robust five‐ and fourfold increase in the RNA levels of HOTAIR‐N in lrECM 3D cultures of MDA‐MB‐231 and Hs578T cells, respectively (Fig. 5C). To determine whether HOTAIR‐N accounted for increase in HOTAIR in lrECM 3D culture, we transfected the HOTAIR‐N‐specific siRNA into MDA‐MB‐231 cells. The RNA levels of total HOTAIR and HOTAIR‐N were substantially reduced by the HOTAIR‐N‐specific siRNA to 40% and 20% (Fig. 5D). Consistently, we observed a 4.7‐fold increase in H3K4me3‐associated HOTAIR‐N promoter (−139 to −247 relative to the transcription initiation site) in Hs578T and MDA‐MB‐231 cells in lrECM 3D culture over 2D culture (Fig. 5E). The increase in H3K4me3 led us to question whether inhibition of histone deacetylases (HDAC) was able to increase HOTAIR expression in 2D culture. Exposure to a pan‐HDAC inhibitor TSA (500 nm, 72 h) increased the RNA levels of HOTAIR to 108‐fold over the DMSO group in MDA‐MB‐231 (Fig. 5F). These findings indicated a correlation between H3K4me3 of the HOTAIR‐N promoter and induction of HOTAIR in lrECM 3D culture of Claudin‐low breast cancer cells.

To determine whether HOTAIR‐N accounts for increased expression of HOTAIR in breast cancer patient biopsies, we surveyed the TCGA Invasive Breast Carcinoma RNA‐Seq data. We selected 14 paired samples that exhibited higher increase in expression of HOTAIR in tumor over their paired normal tissues. We analyzed HOTAIR transcripts by transcript per million (TPM) and percentage of each isoform in the tumor samples using RSEM (Li and Dewey, 2011; Strong *et al*., 2014). In nine tumor samples, HOTAIR‐N exhibited the highest expression among all three isoforms (Table 1). The average contribution of HOTAIR‐N and HOTAIR‐C was that HOTAIR‐N accounted for 57% and HOTAIR‐C accounted for 31.5% of HOTAIR in breast cancer (Table 1). These findings indicated that HOTAIR‐N is a major isoform in invasive breast carcinoma.

**Table 1:** Expression of HOTAIR isoforms in human breast cancer specimens. The TCGA invasive breast carcinoma RNA‐Seq data were surveyed for expression of HOTAIR isoforms using RSEM. Fourteen samples were selected because they exhibited substantial increase in HOTAIR expression in the tumor tissues over their paired normal tissues. Amount of each HOTAIR isoform in each tumor tissue was determined using transcript per million reads (TPM). Percentage of each isoform (IsoPct) in each tumor tissue was also determined. NR_047517 corresponds to HOTAIR‐N. NR_003716 corresponds to HOTAIR‐C

Tumor ID	NR_047517		NR_47518		NR_003716	
	HOTAIR‐N				HOTAIR‐C	
	TPM	IsoPct	TPM	IsoPct	TPM	IsoPct
T73	33.72	91.83	2.46	6.71	0.54	1.46
T25	7.99	90.44	0.63	7.11	0.22	2.45
T85	11.32	90.01	1.02	8.14	0.23	1.85
T87	15.99	75.01	1.79	8.37	3.54	16.62
T46	15.40	69.47	3.44	15.53	3.33	15.01
T102	9.32	67.17	0.88	6.32	3.68	26.51
T54	15.79	62.60	2.34	9.27	7.10	28.13
T71	8.69	57.84	1.23	8.16	5.11	34.00
T110	3.84	48.46	0.57	7.22	3.51	44.32
T77	5.44	42.11	2.90	22.42	4.58	35.47
T80	3.90	39.71	0.60	6.06	5.33	54.23
T19	8.39	29.24	5.13	17.87	15.18	52.89
T98	2.52	19.63	3.01	23.43	7.31	56.94
T2	1.59	14.04	1.73	15.23	8.02	70.72
Average	10.28	56.97	1.98	11.56	4.83	31.47

### 3.4 Requirement of BRD4 for induction of HOTAIR expression in lrECM 3D culture

**Figure 6:**
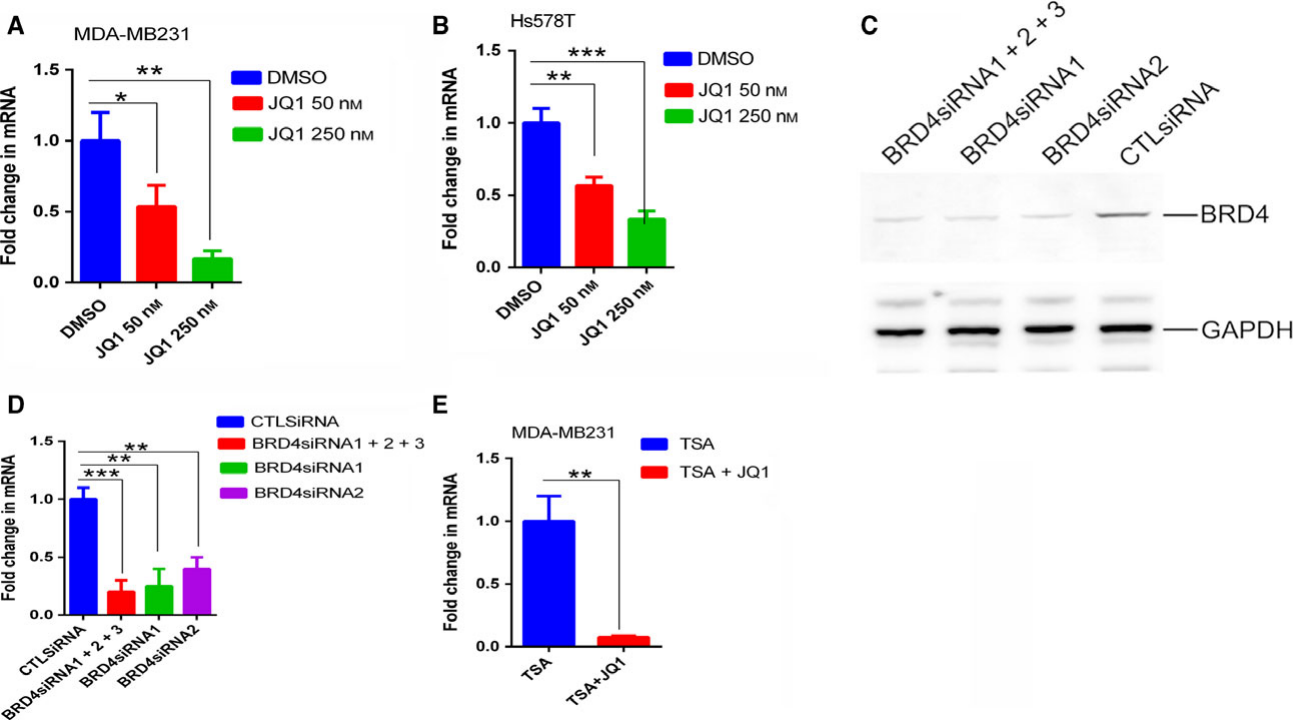
Reduced Expression of HOTAIR by Inhibition of BRD4. (A) Total cell RNA was extracted from lrECM 3D cultures of MDA‐MB‐231 cells treated with either a BRD4‐specific inhibitor JQ1 (50 and 250 nm) or DMSO for 4 days. The RNA levels of HOTAIR were measured using qRT‐PCR. A fold change of the HOTAIR RNA was obtained by normalizing to the housekeeping gene RPLP0 and setting the values from the DMSO‐treated group to one. (B) Similar to part (A) except that the RNA levels of HOTAIR were measured in Hs578T. (C) MDA‐MB‐231 cells were transfected with either BRD4‐specific siRNA (BRDRsiRNA) or control siRNA (CTLsiRNA) and seeded in lrECM 3D culture. Total cell RNA was extracted on day 4 in lrECM 3D culture. The protein levels of BRD4 were assessed using immunoblots. (D) Similar to part (C) except that the RNA levels of HOTAIR were assessed using qRT‐PCR. (E) Total cell RNA was extracted from 2D culture of MDA‐MB‐231 cells treated with either TSA (500 nm) alone or a combination of TSA and JQ1 (250 nm) for 72 h. The RNA levels of HOTAIR were measured as in part (A). *, **, and *** indicated a *P* value < 0.05, 0.01, and 0.001, respectively.

BRD4 binds histone markers for active transcription to promote gene expression (Loven *et al*., 2013; Zuber *et al*., 2011). Because we observed an increase in H3K4me3 associated with the HOTAIR‐N promoter, we speculated that BRD4 mediated induction of HOTAIR in lrECM 3D culture of Claudin‐low breast cancer cells. To test this hypothesis, we treated MDA‐MB‐231 and Hs578T cells in lrECM 3D culture with a BRD4‐specific inhibitor JQ1 for 4 days. JQ1 (50 and 250 nm) substantially reduced the RNA levels of HOTAIR in lrECM 3D culture of both cell lines (Fig. 6A,B). To confirm the requirement of BRD4 for the activation of HOTAIR, we transfected MDA‐MB‐231 cells with the BRD4‐specific siRNA or control siRNA. The protein levels of BRD4 were substantially reduced by BRD4 siRNA (two individual siRNA and a pool of three siRNA) when compared with the control siRNA group (Fig. 6C). HOTAIR RNA levels were also substantially reduced by the BRD4 siRNA when compared with the control siRNA group in lrECM 3D culture (Fig. 6D). We postulated that BRD4 was also required for the activation of HOTAIR by the HDAC inhibitor TSA in 2D culture. We exposed MDA‐MB‐231 cells to TSA alone (500 nm) with or without JQ1 in 2D culture for 72 hrs. As expected, JQ1 (250 nm) reduced the RNA levels of HOTAIR in the presence of TSA to 7% of that in the TSA‐alone group (Fig. 6E).

**Figure 7:**
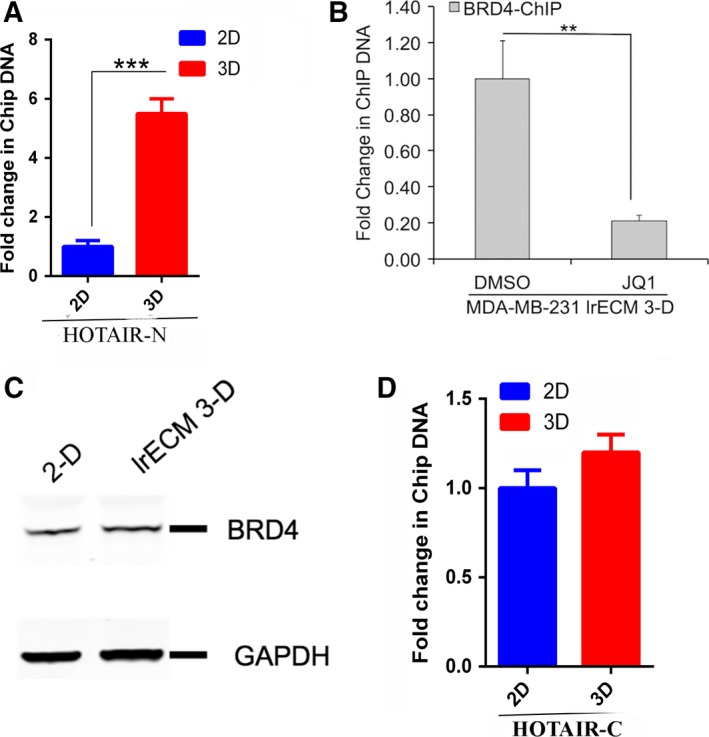
Elevated Binding of BRD4 to the HOTAIR Promoter in lrECM 3D culture. (A) ChIP assays were carried out using a BRD4‐specific antibody in 2D and lrECM 3D cultures of MDA‐MB‐231 cells on day 4. The BRD4‐bound HOTAIR‐N promoter was measured using qPCR and normalized to their corresponding input. A fold change of the BRD4‐bound HOTAIR‐N promoter in lrECM 3D culture over 2D culture was obtained by setting the values from 2D culture to one. (B) Similar to part (A) except that the BRD4‐bound HOTAIR‐N promoter was compared between lrECM 3D cultures treated with either JQ1 (250 nm) or DMSO. (C) Total cell lysates were extracted from MDA‐MB‐231 cells in 2D and lrECM 3D cultures on day 4. The protein levels of BRD4 were measured using immunoblots. (D) Similar to part (A) except that the BRD4‐bound HOTAIR‐C promoter was compared between 2D and lrECM 3D cultures. A fold change of the BRD4‐bound HOTAIR‐C promoter in lrECM 3D culture over 2D culture was obtained by setting the values from 2D culture to one. ** and *** indicate a *P* value < 0.01 and 0.001, respectively.

To determine the association between BRD4 binding and HOTAIR expression, we carried out ChIP assays to compare BRD4 binding to the HOTAIR‐N promoter in 2D and lrECM 3D cultures of MDA‐MB‐231 cells. We observed a 5.5‐fold increase in the BRD4‐bound HOTAIR‐N promoter (−139 to −247 relative to the transcription initiation site) in lrECM 3D culture over 2D culture (Fig. 7A). We then questioned whether JQ1 disrupted BRD4 binding to the HOTAIR‐N promoter because JQ1 inhibited the induction of HOTAIR in lrECM 3D culture (Fig. 6A,B). Indeed, JQ1 (250 nm) substantially reduced the BRD4‐bound HOTAIR‐N promoter to 20% of that in the DMSO‐treated group (Fig. 7B). We examined the expression of BRD4 in lrECM 3D and 2D cultures. The protein levels of BRD4 were comparable between 2D and lrECM 3D cultures of MDA‐MB‐231 cells (Fig. 7C). In contrast, BRD4 binding to the HOTAIR‐C promoter exhibits minimal difference between 2‐D and lrECM 3‐D cultures (Fig. 7D). These data indicated that BRD4 mediated induction of HOTAIR in lrECM 3D culture via increased binding to the HOTAIR‐N promoter.

## 4 Discussion

Herein, we demonstrate that the lncRNA HOTAIR is induced in lrECM 3D culture of Claudin‐low breast cancer cells over conventional 2D culture. Such induction is mediated by epigenetic activation of a novel isoform of HOTAIR.

Claudin‐low breast cancer cells are enriched with the genes that are critical to cellular responses to ECM (Charafe‐Jauffret *et al*., 2009; Prat *et al*., 2010). Therefore, understanding of cancer biology of Claudin‐low breast cancer cells attached to ECM is particularly important. We have reported miRNA that are only differentially expressed between Claudin‐low (MDA‐MB‐231) and luminal A (MCF‐7) breast cancer cells in lrECM 3D culture (Nguyen *et al*., 2012). Our previous report implies a critical role of noncoding RNA in cell responses to ECM. Our current study strengthens and expands this notion. Our results indicate that HOTAIR, a classic example of tumor‐promoting lncRNA, is induced in lrECM 3D culture of Claudin‐low breast cancer cells, and such induction requires the canonical ECM signaling pathway, namely integrins and Src kinase (Figs 1, 2, 3). We are aware that inhibition of integrin α2 and Src kinase results in substantial, but incomplete inhibition of HOTAIR expression in lrECM 3D culture (Figs 2 and 3). These findings likely reflect the complexity in composition of ECM and the signaling pathways in response to ECM. Interference of other integrin receptors and kinases individually and in combination, such as integrin α6 (major receptors for laminin), is still needed to determine the role of each individual ECM and its responsive cellular signaling molecules in the activation of HOTAIR expression (Kenny *et al*., 2007b; Lee *et al*., 2007; Lu *et al*., 2012; Weaver *et al*., 1997; Ziober *et al*., 1999). More importantly, our results indicate that HOTAIR expression is required for invasive growth of Claudin‐low breast cancer cells, particularly cell viability (Fig. 4). These findings likely reflect that a broad spectrum of cellular responses to ECM are regulated by lncRNA.

Our current knowledge of epigenetic regulation of gene expression in breast cancer cells is largely obtained using conventional 2D culture. However, two critical factors, attachment to ECM and three‐dimensional growth, are absent in 2D culture. Our results suggest distinct epigenetic regulation of lncRNA gene expression between 2D and lrECM 3D cultures (Figs 5, 6, 7). As a reader of transcriptionally active histone markers, BRD4 binding is likely a consequence of increased H3K4me3 in the HOTAIR‐N promoter because BRD4 binding was required for the induction of HOTAIR in lrECM 3D culture and TSA‐induced HOTAIR expression in 2D culture (Dey *et al*., 2003). Our results warrant further investigation of epigenetic regulation of gene expression by ECM and three‐dimensional culture in breast cancer cells. The insight obtained from lrECM 3D culture can provide more reliable and accurate guidance for *in vivo* studies of epigenetic mechanisms in breast cancer.

HOTAIR is intensely studied in cancer (Loewen *et al*., 2014). However, its isoforms have not been characterized although aberrant activation of the promoter region upstream of HOTAIR‐N has been implied in breast cancer (Milevskiy *et al*., 2016). Our results indicate that the novel isoform HOTAIR‐N is activated in lrECM 3D culture of Claudin‐low breast cancer cells (Fig. 4). More importantly, a survey of the TCGA invasive breast carcinoma RNA‐Seq data suggests that HOTAIR‐N is the predominant isoform in invasive breast carcinoma (Table 1). Recognition of HOTAIR‐N as a major isoform in breast cancer can initiate several new frontiers. Firstly, HOTAIR‐N is transcribed from the first intron of HOXC11 and forms sense–antisense gene pair with HOXC11 (Fig. 5B). This overlapping implies *in sis* action of HOTAIR in breast cancer besides its established *in trans* action via binding to PRC2 (Gupta *et al*., 2010; Zhuang *et al*., 2015). This notion is appealing because HOXC11 promotes breast cancer and the importance of PRC2 to HOTAIR functions has been challenged recently (McIlroy *et al*., 2010; Portoso *et al*., 2017). Secondly, the HOTAIR‐N‐HOXC11 region contains a CpG island with a CpG count of 160 assigned by the UCSC Genome Browser (Kent *et al*., 2002). The locus presents an ideal platform to investigate dysregulation of a sense–antisense gene pair in the context of a CpG island. This is particularly important because of global dysregulation of sense–antisense gene pairs in breast cancer (Maruyama *et al*., 2012).

## 5 Conclusions

We demonstrate that HOTAIR expression is activated by increased H3K4me3 and BRD4 binding to a novel HOTAIR‐N promoter in Claudin‐low breast cancer cells attached to ECM. Thus, we propose an emphasis on the overlooked interactions between ECM, lncRNA, and epigenetic coding in breast cancer. We also propose a focus on the novel HOTAIR‐N isoform in the overlapping HOTAIR‐HOXC11 locus.

## Author contributions

BS conceived the study and wrote the manuscript. ML, XL, and YZ carried out the experiments. ML analyzed the results and prepared the figures. ZL and EKF analyzed the RNA‐Seq data from TCGA.

## Supporting information


**Table S1.** Sequences of the primers and siRNAs.
