# Supplementary material for: Induction of a novel isoform of the lncRNA HOTAIR in Claudin‐low breast cancer cells attached to extracellular matrix
Source: Mol Oncol. 2017 Oct 30;11(12):1698–710. doi: 10.1002/1878-0261.12133 (PMC5709615; doi:10.1002/1878-0261.12133)
Supplement: Supplementary file 1 — Table S1. Sequences of the primers and siRNAs. [file MOL2-11-1698-s001.docx]

| **Gene** | **Primer Sequences** |
| --- | --- |
| **HOTAIR-T Forward** | **5’-AAATATGGCGGCGTCTACACGGAA-3’** |
| **HOTAIR-T Reverse** | **5’-TCCAGAACCCTCTGACATTTGCCT-3’** |
| **HOTAIR-N Forward** | **5’-GGTCTGGGACAGAAGGAAAG-3’** |
| **HOTAIR-N Reverse** | **5’-AGAGCACCTCCGGGATATTA-3’** |
| **HOTAIR-C-Pro Forward** | **5’-CACTGAGGTCAGCTAGACATTTAC-3’** |
| **HOTAIR-C-Pro Reverse** | **5’-CAGCCTAGGGCAAACATCTC-3’** |
| **HOTAIR-N-Pro Forward** | **5’-GCAGGTTACAGCAGAGGATT-3’** |
| **HOTAIR-N-Pro Reverse** | **5’-GACGGACCGACAAGTGAAA-3’** |
| **HOTAIR-NsiRNA** | **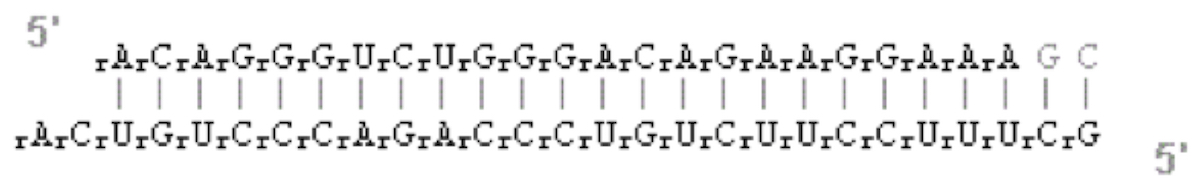** |

**Supplementary Materials Table 1. Sequences of the primers and siRNAs.**
